# Mental illness stigma as a moderator in the relationship between religiosity and help-seeking attitudes among Muslims from 16 Arab countries

**DOI:** 10.1186/s12889-023-16622-7

**Published:** 2023-08-30

**Authors:** Feten Fekih-Romdhane, Suhad Daher-Nashif, Manel Stambouli, Amthal Alhuwailah, Mai Helmy, Hanaa Ahmed Mohamed Shuwiekh, Cheikh Mohamed Fadel Mohamed Lemine, Eqbal Radwan, Juliann Saquib, Nazmus Saquib, Mirna Fawaz, Btissame Zarrouq, Abdallah Y. Naser, Sahar Obeid, Maan Saleh, Sanad Haider, Lahmer Miloud, Manal Badrasawi, Ayman Hamdan-Mansour, Mariapaola Barbato, Aisha Motwakil Bakhiet, Najat Sayem Khalil, Samir Adawi, Fatheya Grein, Alexandre Andrade Loch, Majda Cheour, Souheil Hallit

**Affiliations:** 1 Faculty of Medicine of Tunis, Tunis El Manar University, Tunis, Tunisia; 2 Department of Psychiatry Ibn OmraneThe Tunisian Center of Early Intervention in Psychosis, Razi Hospital, Tunis, Tunisia; 3 School of Medicine, Keele University, Keele, Staffordshire UK; 4 College of Medicine, QU Health, Qatar University, Doha, Qatar; 5 Department of Psychology, Kuwait University, Kuwait, Kuwait; 6 Psychology department, College of Education, Sultan Qaboos University, Muscat, Oman; 7 Psychology department, Faculty of Arts, Menoufia University, Menofia Governorate, Egypt; 8 Department of Psychology, Fayoum University, Faiyum, Egypt; 9 Ministry of Health, Ministry of Health, Nouakchott, Mauritania; 10 Department of Biology, Faculty of Science, Islamic University of Gaza, Gaza Strip, Palestine; 11 College of Medicine, Clinical Sciences Department, Sulaiman AlRajhi University, Bukariyah, Al-Qassim Saudi Arabia; 12 Nursing Department, Faculty of Health Sciences, Beirut Arab University, Beirut, Lebanon; 13 Faculty of Medicine and Pharmacy, Laboratory of Epidemiology and Research in Health Sciences, Fez, Morocco; 14 Department of Applied Pharmaceutical Sciences and Clinical Pharmacy, Faculty of Pharmacy, Isra University, Amman, Jordan; 15 Social and Education Sciences Department, School of Arts and Sciences, Lebanese American University, Byblos, Lebanon; 16 Department of Psychiatry Khobar, Imam Abdulrahman Bin Faisal University, Dammam, KSA Saudi Arabia; 17 Faculty of Medicine and Health Sciences, Behavioral Sciences Dep. A, Aden, Yemen; 18 The National Centre of Research in Social and Cultural Anthropology, Oran, Algeria; 19 Department of Nutrition and Food Technology, Faculty of Agriculture and Veterinary Medicine, An-Najah National University, Nablus, Palestine; 20 School of Nursing, the University of Jordan, Amman, Jordan; 21 Department of Psychology, College of Natural and Health Sciences, Zayed University, Dubai, UAE; 22 Department of Psychiatry, University of Khartoum, Khartoum, Sudan; 23 Psychology Department, Sanaa University, Sanaa, Yemen; 24 College of Medicine and Health Sciences, Behavioural Medicine, Sultan Qaboos University, Muscat, Oman; 25 Sebha Psychiatry Center, Sebha, Libya; 26 Laboratorio de Neurociencias (LIM 27), Faculdade de Medicina, Instituto de Psiquiatria, Hospital das Clinicas HCFMUSP, Universidade de Sao Paulo, Sao Paulo, SP, BR Brazil; 27 Instituto Nacional de Biomarcadores Em Neuropsiquiatria (INBION), Conselho Nacional de Desenvolvimento Cientifico E Tecnológico, Sao Paulo, Brazil; 28 School of Medicine and Medical Sciences, Holy Spirit University of Kaslik (USEK), Jounieh, P.O. Box 446, Lebanon; 29 Research and Psychiatry Departments, Psychiatric Hospital of the Cross, Jal Eddib, Lebanon; 30 Applied Science Research Center, Applied Science Private University, Amman, Jordan

**Keywords:** Stigma, Help-seeking attitudes, Mental illness, Religiosity, Islam, Arab countries

## Abstract

**Background:**

Determining the potential barriers responsible for delaying access to care, and elucidating pathways to early intervention should be a priority, especially in Arab countries where mental health resources are limited. To the best of our knowledge, no previous studies have examined the relationship between religiosity, stigma and help-seeking in an Arab Muslim cultural background. Hence, we propose in the present study to test the moderating role of stigma toward mental illness in the relationship between religiosity and help-seeking attitudes among Muslim community people living in different Arab countries.

**Method:**

The current survey is part of a large-scale multinational collaborative project (StIgma of Mental Problems in Arab CounTries [The IMPACT Project]). We carried-out a web-based cross-sectional, and multi-country study between June and November 2021. The final sample comprised 9782 Arab Muslim participants (mean age 29.67 ± 10.80 years, 77.1% females).

**Results:**

Bivariate analyses showed that less stigmatizing attitudes toward mental illness and higher religiosity levels were significantly associated with more favorable help-seeking attitudes. Moderation analyses revealed that the interaction religiosity by mental illness stigma was significantly associated with help-seeking attitudes (Beta = .005; *p* < .001); at low and moderate levels of stigma, higher religiosity was significantly associated with more favorable help-seeking attitudes.

**Conclusion:**

Our findings preliminarily suggest that mental illness stigma is a modifiable individual factor that seems to strengthen the direct positive effect of religiosity on help-seeking attitudes. This provides potential insights on possible anti-stigma interventions that might help overcome reluctance to counseling in highly religious Arab Muslim communities.

## Introduction

Religiosity is an integral part of most humans' daily lives [1]; particularly in Arab countries (e.g. [2–4],) where Muslims comprise more than 95 percent of the populations [5]. Religiosity may be defined as the set of individuals’ attitudes, behaviors and commitment reflecting respect of higher nonhuman power [6]. Religious beliefs shape the way people view and manage their mental health and illness [7, 8]. This view is originated in the Health Belief Model [9], that counts religion as one of the main variables affecting the way people manage their health and illness in general. Hamdan [10] argue that it is important to consider religious beliefs in relation to mental illness, particularly within Arab societies due to the power of religion (Mostly Islam) within the region over populations. Ng et al. [11] found that individuals who affiliated themselves with a religion (Christianity, Islam, Buddhism/Taoism, and Hinduism) were less likely to seek treatment. They argue that these results reflect beliefs and stigma surrounding mental illness.

Many Arab countries, such as Iraq, Lebanon, Libya, Palestine, Syria, and Yemen, have been experiencing years of wars, armed conflict, and political unrest. The outcomes of these conflicts are expected to result in higher rates of mental health problems, and impairment in the mental health services and programs [12]. The Islamic teachings, which are dominant in most Arab countries, mandate Muslims to seek treatment when they get sick. Unfortunately, Gearing et al. [13] reported that the stigma attached to mental illness is one of the key factors that negatively influences Arab patients with mental illness accessing psychiatric services, contributing to the underutilization of mental health care.

The World Health Organization [14] defines stigma as “a mark of shame, disgrace or disapproval which results in an individual being rejected, discriminated against, and excluded from participating in a number of different areas of society”. The tendency to stigmatize appears to be a profoundly rooted attitude in human nature as a way of responding to people who seem or perform differently. Stigmatization is thereby based on the fear that those who seem different may behave in threatening or unpredictable ways [15]. Religious beliefs have been shown to significantly impact individuals’ attitudes toward mental disorders, however in diverse ways [16]. A systematic review has, for example, reported mixed findings with respect to the relationship between religiosity and stigma in Black Americans [17]. Most of the evidence on the association between religiosity and mental health stigma has emerged from Western countries (Europe and USA) [18–22], while only a very few studies have been performed in Arab countries. For instance, a Jordanian study published in 2021 found that higher levels of religiosity significantly correlated with lower mental health stigma among students in secondary school, but this correlation was no longer significant when adjusting for other sociodemographic variables [23]. Religiosity has also been found to significantly correlate with more positive attitudes toward people with mental illnesses in Muslim Jordanian students [24]. It is widely common in Arab Muslim culture to believe that mental illness is caused by lack of faith [25]. A study found that a large proportion of Qatari Muslim university students agreed that “mental illness is a punishment from God", which was reflected in their high endorsement of stigmatizing attitudes toward people with mental illness [26]. Another study found that 67.3% the of people from the Saudi general population believed that depression was caused by lack of faith and 56% believed in faith healers as an appropriate treatment approach [27]. Additionally, in a study on perceptions of and attitudes toward mental illness among both medical students and the general public in Oman, Al-Adawi et al. [28] found that groups believed that mental illness is caused by spirits and rejected genetics as a significant factor. As in Saudi Arabia, the social stigma surrounding mental issues appears to be profound, and to directly affect help-seeking behavior [29]. On the other hand, there is sufficient evidence that Arab people tend to hold negative attitudes toward professional help-seeking [30–37], and rather tend to rely on informal sources of help rather than seeking mental health care service [38–40]. Indeed, both Arabs living in Arab countries (e.g. [41–44],) and Arab immigrant minorities living in Western countries (e.g. [45–47],) have been found to highly endorse supernatural/religious causal attributions, and thereby tend to prefer seeking help from traditional healers and religious authorities when experiencing mental health problems. In Arab Muslim societies, mental illness is framed and explained through religious beliefs [10]. These beliefs include Punishment from God, a test from God, Jinn, Evil eye, satanic power and more [10, 44]. For instance, Bener and Ghuloum [48] found that almost half of the participants from the general Qatari population, believed that mental illness is a punishment from God, and almost 40% of respondents from believed that people with mental health disorders are “mentally retarded”. A recent systematic literature review identified mental Illness conceptualization, stigma, traditional healing methods, and religious leaders as amongst the major reasons for negative help-seeking attitudes and reluctance to engage in counselling among citizens of the Arab region [36]. A study conducted in Baghdad revealed that 83% of the participants believe that mental illnesses need medical management while 17% trust in traditional methods (religion and faith healers) [49]. Therefore, help-seeking attitudes, intentions and behaviors appear to be multi-determined in nature [50]; and there seems to be a complex interplay between religiosity, stigma, and help-seeking.

Many studies have addressed mental health stigma and its impact on help seeking behaviors. Some of these studies were conducted in the Arab region (e.g. [31, 37, 51, 52],) where religion was mentioned as a major contributing factor in shaping perceptions and social attitudes towards mental illness. In 2001, the WHO identified mental health stigma as a key barrier to effective treatment of mental illness due to its negative impact on individuals’ willingness to seek treatment. In their systematic review of 144 studies and 90189 participants, Clement et al. [53] reported that the median association between stigma and help-seeking was d =  − 0.27, with internalized and treatment stigma being most often associated with reduced help-seeking. They also reported that mental health stigma was the fourth highest ranked barrier to help-seeking [53]. Similarly, other systematic reviews and meta-analyses findings revealed that individuals’ own stigmatizing attitudes toward mental illness (or perceived public stigma) were associated with a significant 0.82-fold decrease in active help-seeking [54]; albeit anti-stigma interventions showed no effectiveness in improving formal help-seeking behaviors in the general public [55]. This suggests that variables other than stigma should be taken into account when assessing factors driving help-seeking in the community.

To date, scarce studies have focused on factors related to help-seeking in low- and middle-income countries [55], with the Middle East and North Africa (MENA) region as no exception. To the best of our knowledge, no previous studies have examined the relationship between religiosity, stigma and help-seeking in an Arab Muslim cultural background. Choosing religious and traditional healers as first care providers over health care professionals may lead to substantial delays in accessing formal mental health services [56]. Thus, determining the potential barriers responsible for delaying access to care, and elucidating pathways to early intervention should be a priority, especially in Arab countries where mental health resources are limited [57]. Hence, we propose in the present study to test the moderating role of stigma toward mental illness in the relationship between religiosity and help-seeking attitudes among Muslim community people living in different Arab countries.

## Methods

### Sample and procedure

The current survey was part of a large-scale multinational collaboration project (StIgma of Mental Problems in Arab CounTries [The IMPACT Project]) [58]; aimed at providing a cross-cultural examination of stigma towards patients with mental illness across the Arab countries in the Middle East and North Africa (MENA) region. We carried-out a web-based cross-sectional, and multi-country study between June and November 2021 (Further details about the project have been reported elsewhere [44]). Eligible participants were community individuals aged over 18 years, Arabic-speaking and Muslim. Data collection in all countries was performed using an anonymous online questionnaire (in the Arabic language) and convenience sampling. Invitations to participate in the study were sent through social media platforms and acquaintances; after that, we used the snowball sampling technique (each subject provided multiple referrals) to recruit the rest of the sample. No credits were awarded for participating.

Initially, 10036 valid responses have been received. A total of 254 participants were excluded because of their religious affiliation (Christianity 48.0%, Atheism 41.3%, Judaism 2,8%, other religions 7.9%). The final sample comprised 9782 Arab Muslim participants originating from and residing in 16 Arab countries. The distribution of participants across countries was as follows: Algeria: *N* = 150; Egypt: *N* = 1029; Jordan: *N* = 426; Kingdom of Saudi Arabia: *N* = 872; Kuwait: *N* = 2182; Lebanon: *N* = 781; Libya: *N* = 108; Mauritania: *N* = 396; Morocco: *N* = 328; Oman: *N* = 78; Palestine: *N* = 448; Qatar: *N* = 130; Sudan: *N* = 102; Tunisia: *N* = 2343; United Arab Emirates: *N* = 150; and Yemen: *N* = 259.

As for ethical considerations, the study was performed according to the Declaration of Helsinki for human research, and approved by the Ethics Committee of the home institution of the Principal Investigator, FFR (Razi Psychiatric Hospital, Tunisia) [Reference number is 2021–0034]. All participants provided their online informed consent to participate before beginning the survey. No compensation was offered.

### Measures

We collected the following sociodemographic data for all participants: age, gender, marital status (married vs. unmarried [single, divorced/separated, widowed]), education level (primary, secondary, tertiary), self-perceived socioeconomic status (high, average, low), residency (urban, rural), as well as family and personal psychiatric history (any mental illness diagnosed by a professional; yes/no). Information on mental health stigma, religiosity and help-seeking attitudes was collected using the Arabic versions of the following measurement instruments:

### The community attitudes toward the mentally ill scale (CAMI) [59, 60]

The CAMI is a measure evaluating public attitudes towards people with mental illness through forty items and four subscales: Social Restrictiveness *(e.g.,* “*The mentally ill should not be given any responsibility)*, Benevolence *(e.g., The mentally ill have for too long been the subject of ridicule”)*, Authoritarianism (e.g., “*One of the main causes of mental illness is a lack of self-discipline and will power”)*, and Community Mental Health Ideology (e.g., “*As far as possible, mental health services should be provided through community based facilities”*). Each item is rated on a five-point Likert scale ranging from 1 (strongly agree) to 5 (strongly disagree). Lower total scores indicate more stigmatizing attitudes toward mental illness. The Cronbach’s alpha for the total scale in the present study was of 0.875.

### The attitudes toward seeking professional psychological help scale-short form (ATSPPH-SF) [30, 61]

The ATSPPH-SF is a 10-item four-point Likert scale *(e.g., “If I believed I was having a mental breakdown, my first inclination would be to get professional attention”; “I would want to get psychological help if I were worried or upset for a long period of time”).* Total scores range from 0 to 40, with higher total scores referring to greater positive attitudes toward help-seeking (Please refer to [44] for further details about means and standard deviations of stigma [CAMI] and help-seeking attitudes [ATSPPH-SF] by country). In the current study, the Cronbach's alpha value was of 0.69, indicating an acceptable overall internal consistency.

### The Arabic religiosity scale (ARS) [62]

The ARS is a five-item measure that assesses participants’ religiosity levels in three dimensions: (1) Behavioral religiosity (private and public) *(e.g., “Do you have individual religious activities (individual prayers)?”)*, (2) Cognitive/affective importance of religiosity (both at lifetime and at time of difficulties) *(e.g., “What is the importance of religious beliefs in the full curriculum of your life?”)*, and (3) General self-rate level of belief *(e.g., “How do you evaluate the degree of your faith?”)*. Example of items: Answers to each item vary from 1 (never, absent, not important) to 4 (always, most times, great importance). Only total scores have been considered in the present study, with higher scores indicating greater levels of religiosity. The present sample revealed a Cronbach’s alpha of 0.70 for the ARS total score.

### Statistical analysis

The SPSS software v.25 was used for the statistical analysis. The CAMI, ATSPPH-SF, and ARS scores were considered normally distributed since the skewness and kurtosis values varied between -2 and + 2. The Student t was used to compare two means and the Pearson test was used to correlate two continuous variables. The moderation analysis was conducted using PROCESS MACRO v3.4, model 1 taking religiosity as the independent variable, stigma as the moderator and help-seeking attitudes as the dependent variable. Results were adjusted over variables that showed a *p* < 0.25 in the bivariate analysis. *P* < 0.05 was deemed statistically significant.

## Results

### Sociodemographic and other characteristics of the sample

A total of 9782 were involved in this study, with a mean age of 29.67 ± 10.80 years and 77.1% females. Other descriptive statistics of the sample can be found in Table 1.

**Table 1:** Sociodemographic and other characteristics of the sample (*N* = 9782)

Variable	N (%)
Sex	
Male	2236 (22.9%)
Female	7546 (77.1%)
Marital status	
Unmarried	5719 (58.5%)
Married	4063 (41.5%)
Education level	
Secondary or less	1032 (10.5%)
Tertiary	8750 (89.5%)
Self-perceived socioeconomic status	
High	2390 (24.4%)
Average	6761 (69.1%)
Low	631 (6.5%)
Residency	
Urban	8378 (85.6%)
Rural	1404 (14.4%)
Personal psychiatric history	
No	8806 (90.0%)
Yes	976 (10.0%)
Personal medical history	
No	8856 (90.5%)
Yes	926 (9.5%)
	**Mean ± SD**
Age (years)	29.67 ± 10.80
CAMI score	133.59 ± 17.64
Help-seeking attitudes	21.80 ± 6.02
Religiosity	15.28 ± 2.66

### Bivariate analysis of factors associated with help-seeking attitudes

The results of the bivariate analysis of factors associated with help-seeking attitudes are summarized in Table 2. The results showed that a higher mean help-seeking attitudes score was found in females, participants with a university level of education, with a good socioeconomic status, with a personal psychiatric or medical history. Higher CAMI and religiosity scores were significantly associated with higher help-seeking attitudes scores.

**Table 2:** Bivariate analysis of factors associated with help-seeking attitudes (ATSPPH-SF total scores)

Variable	Categorical variables	
	**mean ± SD**	***p***
Sex		< .001
Male	21.31 ± 6.25	
Female	21.95 ± 5.94	
Marital status		.067
Single	21.89 ± 6.26	
Married	21.67 ± 5.66	
Education level		.003
Secondary or less	21.27 ± 6.04	
University	21.86 ± 6.01	
Socioeconomic status		< .001
Good	22.08 ± 6.55	
Average	21.80 ± 5.79	
Bad	20.73 ± 6.20	
Region of living		.631
Urban	21.79 ± 5.93	
Rural	21.88 ± 6.53	
Personal psychiatric history		< .001
No	21.70 ± 5.98	
Yes	22.74 ± 6.29	
Personal medical history		.004
No	21.86 ± 5.99	
Yes	21.23 ± 6.26	
	**Continuous variables**	
	***r***	***p***
Age	-.003	.737
CAMI	.27	< .001
Religiosity	.07	< .001

*CAMI* Community Attitudes toward the Mentally Ill scale, *ATSPPH-SF* Attitudes Toward Seeking Professional Psychological Help Scale-Short Form

### Moderation analysis with help-seeking attitudes scores taken as the dependent variable

**Fig. 1:**
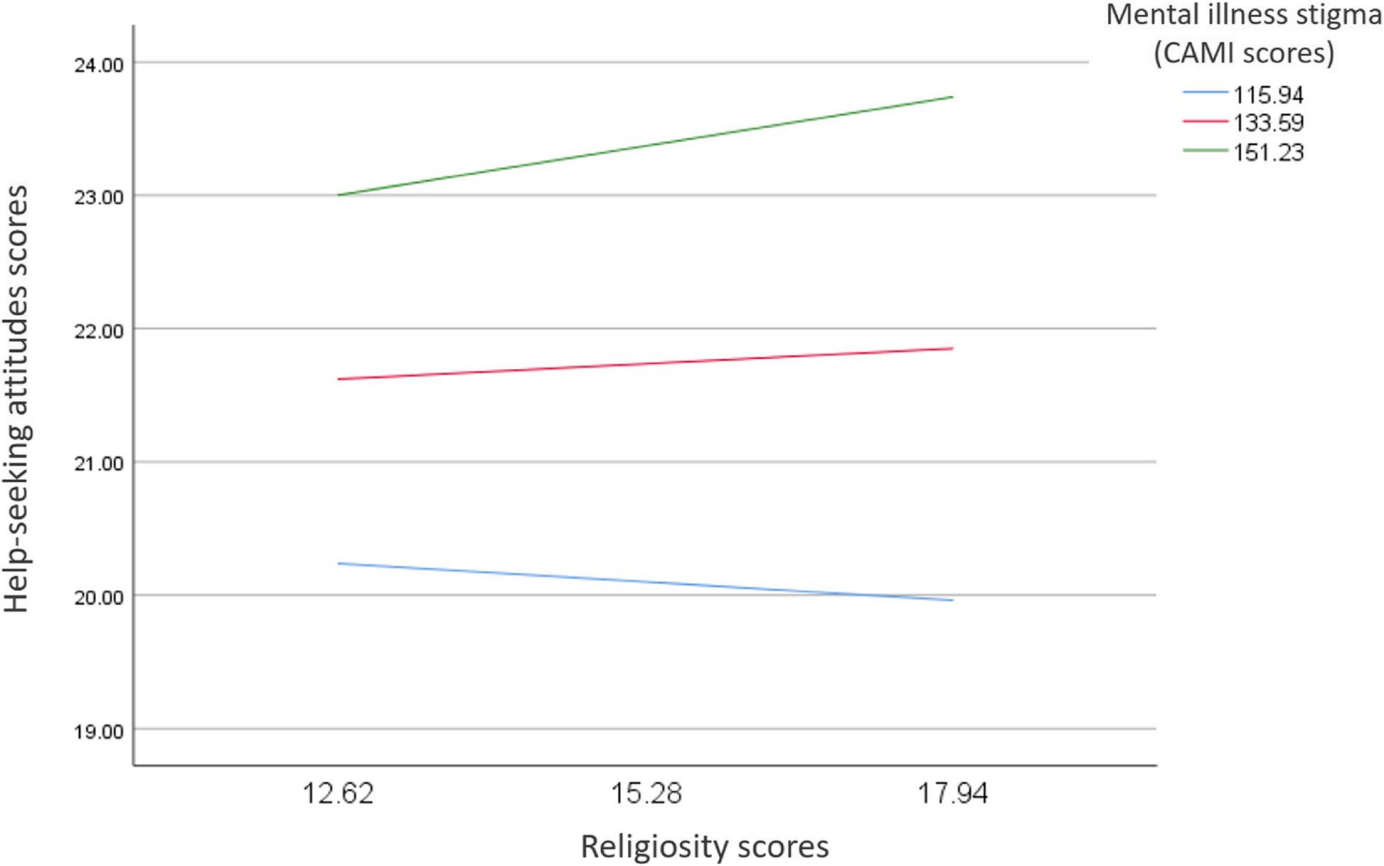
Graphical depiction of the association of the interaction religiosity by mental illness stigma and help-seeking attitudes. Low and high levels of mental illness stigma were plotted at -1 SD and + 1 SD respectively

The details of the moderation analysis are summarized in Tables 3 and 4 and Fig. 1. The interaction religiosity by CAMI was significantly associated with help-seeking attitudes (Beta = 0.005; *p* < 0.001); at high levels of CAMI, higher religiosity (Beta = 0.14, *p* < 0.001) as significantly associated with more favorable help-seeking attitudes.

**Table 3:** Moderation analysis taking religiosity as the independent variable, stigma (CAMI total scores) as the moderator and help-seeking attitudes as the dependent variable

Moderator	Beta	t	*p*	95% CI
Religiosity	-.68	-4.07	< .001	-1.00; -.35
CAMI	.01	.53	.594	-.03; .05
Interaction religiosity by CAMI	.005	4.28	< .001	.003; .008

Results adjusted over gender, marital status, education level, personal history of medical illness, personal history of psychiatric illness, and socioeconomic status. *CAMI* Community Attitudes toward the Mentally Ill scale

**Table 4:** Conditional effects of the focal factors at values of CAMI as the moderator

	**Beta**	**t**	***p***	**95% CI**
115.94	-.05	-1.75	.080	-.11; .01
133.59	.04	1.85	.065	-.003; .09
151.23	.14	3.97	< .001	.07; .21

*CAMI* Community Attitudes toward the Mentally Ill scale

It is noteworthy that the interactions community mental health ideology by religiosity (Beta = -0.001; *t* = -0.42; *p* = 0.674; 95% CI -0.01, 0.01), authoritarianism by religiosity (Beta = -0.007; *t* = -1.46; *p* = 0.145; 95% CI -0.02, 0.002), benevolence by religiosity (Beta = -0.007; *t* = -1.45; *p* = 0.148; 95% CI -0.02, 0.002) and social restrictiveness by religiosity (Beta = 0.002; *t* = 0.56; *p* = 0.578; 95% CI -0.01, 0.01) did not show a significant association with help-seeking attitudes.

## Discussion

In this study we sought to provide, for the first time, an in-depth examination of the relationship between religiosity and help-seeking attitudes in Arab Muslim community people, by investigating the moderating effects of mental health stigma in this relationship. Findings revealed that higher religiosity levels were associated with greater positive attitudes towards seeking professional help. In addition, moderation analyses were significant, showing that the strength of the relationship between religiosity and help-seeking attitudes was strongly influenced by mental health stigma. We discuss the relevance and implications of these results later in this paper.

Regarding the direct effect, higher religiosity was significantly linked to more favorable attitudes toward seeking formal professional help. This further supports that religion “can act as such a dynamic social force”, and should be accounted for when studying human psychology, perception and behavior [16]. While some claim that religious people from Muslim countries rely on religious resources for treatment such as Quran reciting (e.g., [63]), our findings show that religiosity might be a factor for better perceiving help-seeking. Any comparisons with previous literature in Muslim communities are challenging, given that most of the previous studies in this area have investigated religious factors as causal attributions through qualitative methods or self-developed measures (e.g. [26, 64–68],), whereas no studies have used a valid measure to assess the religiosity construct specifically in relation to help-seeking. Besides, while a substantial amount of literature has consistently found that religiosity is protective against mental health problems [69], only dearth of research examined the relationship between religiosity and help-seeking [70]. Consistent with our findings, a study reported that higher levels of private, non-organizational religiosity were correlated with greater utilization of professional mental health services among American community older adults [71]. Another study suggested that this positive effect of religiosity on counseling could differ depending on individuals’ level of distress [72]. Furthermore, a recent Lebanese study [73] showed a positive correlation between higher levels of mature religiosity and higher engagement coping strategies but less disengagement strategies. This association might be explained by the belief that God is in control of the problem [74]; the individual feels more encouraged to face the problem or emotion because they believe that God can alter the situation for the better. It promotes engagement, like seeking-help, especially when there are high chances of achieving the established goals, and, to a lesser extent, disengagement, which is more likely to be adaptive, especially under adverse circumstances [75]. Contrarily, a large study among African American adults showed inverted patterns of associations between religiosity and service use [76]. In Islam, seeking treatment for mental health issues does not conflict with seeking help from God [77], which might explain our results. Beyond this direct effect, we explored the moderating role of mental illness stigma in the relation religiosity-help-seeking attitudes, which we discuss in the next section. Investigating moderators may help elucidate the nature of this relationship and provide explanations for the variations in results across studies.

Moderation refers to a situation in which a moderator changes either the strength or the direction of a relationship between two constructs; the relationship is thus not constant but depends on the values of the moderator variable [78]. As expected, we found that both mental health stigma had a significant moderating role in the path from religiosity to help-seeking attitudes. This means that the relationship between religiosity and help-seeking attitudes differs as a function of stigmatizing beliefs towards mental illness. More precisely, mental illness stigma has a pronounced negative effect on the religiosity to help-seeking attitudes relationship – the higher the stigma toward mental illness (lower CAMI score), the weaker the relationship between religiosity and help-seeking attitudes. Additionally, in lowly and moderately stigmatizing individuals, religiosity was positively associated with help-seeking attitudes, whereas for highly stigmatizing individuals, religiosity was negatively associated with help-seeking attitudes. This finding is consistent with previous literature stipulating that people who tend to exhibit lower levels of mental health stigma have more favorable attitudes toward professional help-seeking [54]. People that are more religious tend to more strongly perceive counseling as appropriate and effective when they display low stigmatizing attitudes toward mental illness. These findings suggest that tackling stigmatizing attitudes toward mental illness held by community individuals may help strengthen the positive effects of religiosity on help-seeking attitudes.

### Limitations

Some limitations should be discussed. Because of the cross-sectional design, we could not conclude about causality, and longitudinal research is still needed to determine directionality in the associations between study variables. In addition, while we report on findings among Arab Muslims, we cannot catch the impact of culture and how this interacts with religiosity. For sorting this, a future study on Muslim communities in other regions (such as South Asia, Turkey, and Iran) can report the impact of culture on Muslim communities. Furthermore, removing other religions from the study at its beginning, might limit us from reporting on differences between different religions. For solving this, a future study among bugger sample of non-Muslim Arabs, can tell about the specific impact for religion, because they all affiliate to the same culture. In addition, although Arab populations live in the MENA share values, beliefs, and traditions [42, 79], there are some differences between the different three main regions (Middle East, North Africa and the Gulf States) that we did not report in this study because we focused on religion rather than culture. These differences might have influenced attitudes toward mental illness and help-seeking across countries [80]. Future research that focuses on culture supposed to highlight these differences, and how these differences shape mental health stigma and help seeking behaviors. Finally, our study did not assess the dimension of self-stigma, hence, it’s recommended to assess in future studies the four stigma types (help-seeking attitudes and personal, self and perceived public stigma) on active help-seeking in the general population.

### Study implications

The present study revealed that total religiosity (involving behavioral, affective and general level of belief) is positively associated with favorable attitudes toward help-seeking in a large sample of Muslim people from different Arab countries. These findings may change our current approach to addressing reluctance towards help seeking and our perception that religiosity is regarded as a barrier to care access in Arab Muslim contexts, while it could act as a facilitator in certain circumstances. Longitudinal studies are necessary before drawing any firm conclusions. Interestingly, this study revealed that highly religiosity is associated with favorable attitudes toward seeking professional help in individuals who exhibited low to moderate levels of stigma toward mental illness. This finding suggest that mental illness stigma is a modifiable individual factor that seems to strengthen the direct positive effect of religiosity on help-seeking attitudes. This provides potential insights on possible anti-stigma interventions that might help overcome reluctance to counseling in highly religious Arab Muslim community individuals. Stigma is universal, occurring in every country and region of the world, albeit with different manifestations in different social and cultural contexts [81]. Given the uniqueness of the cultural Arab context, there is a strong need to design culturally- appropriate and sensitive interventions intended to reduce the occurrence and impact of stigma related to mental illness, in order to maximize their efficiency [82, 83]. At present, there is insufficient evidence on the types of intervention that may be effective and feasible in low- and middle-income countries [84]. A very few anti-stigma campaigns have previously been implemented in some Arab countries, such as the anti-stigma initiative launched by the World Psychiatric Association (WPA) in Morocco and Egypt [85]. Besides, there have been some initiatives to suggest avenues to reduce stigma and combat negative stereotype related to mental illness in the Middle East; such as a systematic literature by Sewilam et al. [42], which proposed some lines of intervention (educating family caregivers and young people in schools, increasing cooperation between medical professionals and traditional healers). A qualitative review of literature suggested that implementing mental health legislations and policies within the health-care setting might be effective in combatting stigma in Arab countries [38]. More efforts should be put on developing and implementing evidence‐informed population-based anti-stigma programs that are carefully tailored to the Arab Muslim cultural backgrounds. Finally, additional international and cross-cultural research is required to further elucidate the specific mechanisms (moderators and mediators) by which religiosity may contribute to positive or negative attitudes toward help-seeking.

## Conclusion

Religion has a strong impact on Muslim communities, and religious beliefs can be major factors in shaping health perceptions and behaviors. To date, there has been scant or no research attention to the relationship between religiosity and help-seeking attention in Arab Muslim contexts. Despite a general agreement that religious factors are closely linked to negative mental help-seeking attitudes and reported avoidance of help-seeking, the present findings suggest for the first time the contrary. We also demonstrated that mental illness stigma is a modifiable individual factor that seems to strengthen the direct positive effect of religiosity on help-seeking attitudes. The lower the stigma toward mental illness, the stronger the relationship between religiosity and favorable help-seeking attitudes. These findings may help inform the development of culturally tailored anti-stigma interventions.

## Data Availability

All data generated or analyzed during this study are not publicly available due the restrictions from the ethics committee. Reasonable requests can be addressed to the corresponding author.
